# Direction-of-arrival estimation of multipath signals using independent component analysis and compressive sensing

**DOI:** 10.1371/journal.pone.0181838

**Published:** 2017-07-27

**Authors:** Lin Zhao, Jian Xu, Jicheng Ding, Aimeng Liu, Liang Li

**Affiliations:** College of Automation, Harbin Engineering University, Harbin, Heilongjiang, China; Nanjing Normal University, CHINA

## Abstract

Multipath signal is often considered an interference that must be removed. The coherence between multipath and direct component makes it difficult to use conventional direction-of-arrival (DOA) estimation methods in a smart antenna system. This study demonstrates a new multipath signal DOA estimation technique. Unlike the common methods, without decoherence preprocessing, the proposed algorithm first apply a complex fast independent component analysis (cFastICA) algorithm to obtain the steering vectors with multipath information that corresponds to each source signal. Then, according to the special structure of the obtained steering vectors and spatial sparsity of the multipath signal components, the algorithm uses the solution of the sparse signal reconstruction problem in the compressive sensing (CS) theory, and the DOA estimation of the multipath signal is translated into an l1 norm minimization problem. Finally, we search the space spectrums to acquire the DOAs for each direct component and multipath component. Comparative simulation tests and analysis prove the effectiveness of the proposed algorithm in estimation accuracy in underdetermined conditions.

## 1. Introduction

Array signal processing is widely used in radar, sonar, and seismic exploration, anti-jamming and wireless communications. The global navigation satellite system (GNSS) has received a great deal of attention and become increasingly popular because of its various applications in both civil and military areas. For GNSS anti-jamming, array signal processing techniques can transform the spatial direction of the beam, restrain the interference of the other directions, and transform the signal gain at different spatial positions. By determining the spatial spectra of the impinging targets, the direction-of-arrival (DOA) estimation on multi-antenna GNSS receivers is an important aspect. It uses the relationship between various elements of the sensor array in space to estimate the parameters of the spatial signals.

The signal broadcasted by the GNSS satellite is a spread spectrum signal. The blockage, reflection and diffraction of signals by buildings or other obstacles significantly degrade the availability and accuracy of the GNSS. The reflected signals can interfere with the reception of the signals that are directly received from the satellites; this phenomenon is known as multipath interference or multipath because the signals are received via multiple paths. These multipath interferences must be mitigated or may cause the signal received by the array antenna to have a direct component and several multipath components, which are coherent to one another. These components will be merged into a source signal and make the signal subspace rank deficient when general DOA estimation methods are used, such as multiple signal classification (MUSIC) [1] and estimating signal parameters via rotational invariance techniques (ESPRIT) [2] to find the direction of the multipath signal (the signal with multipath components). The estimated performance will decline and may fail.

For the DOA estimation of multipath signals, most researchers preprocess the coherent signals to achieve decoherence and then estimate the DOAs. Currently, coherent signal-preprocessing methods mainly include the following two categories:

Examples of dimensionality reduction methods are the forward spatial smoothing (SS) algorithm [3], forward and backward spatial smoothing algorithm [4], and modified spatial smoothing algorithm [5]. This type of method is easy to implement, and when combined with the MUSIC algorithm, it is considered a very effective DOA estimation algorithm for multipath signals [6]. However, because the algorithm obtains the decoherence capacity at the expense of loss array aperture and requires more antenna array elements to handle the same number of signals, the estimated performance decreases.

Examples of non-dimensionality reduction methods are the Toeplitz algorithm [7] and subspace fitting algorithm [8]. Toeplitz algorithm is easy to implement and can be used for a low SNR; however, the computation complexity of the algorithm is high, and the estimation error is large. Similar to MUSIC and ESPRIT, the subspace fitting algorithm can be considered a subspace algorithm and has decoherence properties and good estimation performance with a low signal-to-noise ratio and low sampling number. However, the algorithm involves a high-dimensional optimization problem and high computational complexity during the fitting process, and the global convergence of parameters is not guaranteed.

Sparse signal reconstruction in the compressed sensing (CS) theory [9], which is a new method of signal analysis, can be used to obtain a concise expression of the signal and has been used in many fields [10][11][12][13]. Because there are only a few non-zeros in the spatial spectrum of array signals, which represent their corresponding spatial locations, this sparsity can be applied to the DOA estimation. Bilik proposed to use the compressive sensing (CS) theory to reconstruct the high-resolution spatial spectrum from a small number of spatial measurements [14]. Compressive MUSIC identifies the parts of support using CS, after which the remaining supports are estimated using a new generalized MUSIC criterion that can approach the optimal l0-bound with a finite number of snapshots [15]. Li introduced CS to single-snapshot DOA estimation [16]. The method proposed by Wei Zhu provided high resolution while using a uniform linear array without restricting the requirements of the spatial and temporal stationary and correlation properties of the sources and noise [17].

The independent component analysis (ICA) algorithm is also suitable for processing array signals. It has no special requirements for the unknown source signal, noise or transmission channel. The algorithm can separate the mixed signal and has good denoising performance without compromising the details of other signals. Chang proposed a complex-valued ICA approach to simultaneously run the DOA estimation and Blind signal separation [18]. Jančovič presented an underdetermined DOA estimation algorithm that used the ICA and time-frequency masking [19]. The proposed method in [20] extracts the Gaussian noise basis vector using the modified complex-valued FastICA algorithm and improves the resolution capability of a sensor array on which non-Gaussian signal sources with high correlation are impinging. A new method for the DOA estimation of uncorrelated and coherent signals was proposed in [21].

Most DOA estimation methods based on CS and ICA are only used to process the array signal without multipath. As with many decoherence algorithms, the algorithms in [20] and [21] eliminate only the coherence among the signals, obtain the DOA of the direct component, and do not use the coherence information to obtain the DOAs of multipath components.

In this paper, according to the special structure of the steering vector with multipath information, an algorithm based on CS and ICA is proposed. The new method uses the ICA algorithm to obtain the steering vector, which contains the multipath component information, and the CS theory is used to estimate the DOA of the direct component and each multipath component of the array signals. This method is significant to suppress the multipath interference in the beamforming process. In addition, without decoherent preprocessing, the calculation process is simplified.

The structure of the paper is as follows. In Section 2, the signal model with multipath components is introduced. In Section 3, we describe the process using the new approach to achieve the DOA estimation of the multipath signal. In Section 4, some comparable simulation and test results are demonstrated for the proposed method and spatial smoothing with the MUSIC algorithm in different conditions. Section 5 summarizes the conclusions.

## 2. Problem Formulation

Consider a uniform linear array (ULA) with M isotropic sensors and take the first sensor as the reference. N source signals are received by the array antenna and receive a mixed signal of M parallel channels. The result is expressed as

X(t)=AS(t)+n(t) (1)

where *X*(*t*) = [*x*_1_(*t*), *x*_2_(*t*), … *x*_*m*_(*t*)]^T^ is the received signal of each array element; *A* = [*a*_1,_*a*_2,_…*a*_N_] is the direction matrix of the antenna array, which is an array manifold; *a*_*n*_ is a steering vector of the *n*-th channel, whose value is determined by the DOA and array element arrangement in the antenna array; *S*(*t*) = [*s*_1_(*t*), *s*_2_(*t*), … *s*_*N*_(*t*)]^T^ is the source signals; and *n*(*t*) is an added white Gaussian noise (AWGN) vector.

For every source signal, if *P* multipath components are also received, since the multipath components are attenuated relative to the direct component without waveform changing, *a*_*n*_ is rewritten as

$${\hat{a}}_{n}=a_{n}+\sum_{p=2}^{P}c_{nL_{P}}a_{nL_{P}},(n=1,2\cdots N)$$ (2)

where $a_{nL_{p}}$ is the steering vector of the *p*-th multipath component in the *n*-th channel; and $c_{nL_{p}}$ is the propagation attenuation coefficient of the *p*-th multipath component in the *n*-th channel.

The following relationship exists between the angle of arrival *θ*_*n*_ and the steering vector *a*_*n*_ for a ULA:

$$a_{n}={\left[1,e^{j2\pi d\text{sin}\theta_{n}/\lambda },\cdots ,e^{j2\pi (M-1)d\text{sin}\theta_{n}/\lambda }\right]}^{T},(n=1,2\cdots N)$$ (3)

where *d* is the array element spacing, and *λ* is the wavelength of the received signal.

Eq (1) shows that the source signals are linearly mixed by the antenna array and that the array manifold *Â* is an aggregate of the steering vector *â*_*n*_ of each source signal. For the multipath receipt condition, the steering vector also contain multipath information, as expressed in Eq (2), which is the sum of all steering vectors multiplied by their attenuation coefficients.

## 3. Proposed DOA estimation method

Many studies have verified that the mixed signal can be separated into source signals using the ICA algorithm [22]. Hence, the linear mixed array, which is array manifold *Â*, can be obtained. For a ULA, its array manifold follows the Vandermonde matrix structure, and the DOAs can be easily determined [18].

However, in the multipath receipt environments, the steering vector *â*_*n*_ in the array manifold, which is obtained with signal separation using ICA, contains multipath components. These multipath components are included in Eq (4). The steering vector of each component should be resolved in underdetermined conditions. If the expected DOAs are expended to the entire spacing spectrum, the DOA spacing distribution is notably sparse and satisfies the important assumption of the compressive sensing theory. Thus, the sparse spectrum of *â*_*n*_ is obtained by minimizing the *l*_1_ norm using compressive sensing. Then, the expected DOAs are obtained.

Currently, the DOA estimation process can be divided into two steps: separation of the signal using ICA to estimate the array manifold *Â* and obtaining the DOAs of the direct and multipath components using compressive sensing.

### 3.1 Separating signals using the ICA algorithm

The signals from different sources are typically statistically independent from one another. The ICA algorithm uses this characteristic to estimate the source signals from the observed mixed signals.

To use the ICA algorithm, the statistical properties of the source signals satisfy the following assumptions:

1. All the source signals are independent from each other. In practical, this assumption is not strict and easy to satisfy.
2. At most only one of the independent signals can be Gaussian. Most of the digital communication signals can be considered as sub-Gaussian and therefore this assumption holds[23].

Because noise can be used as sources and separated from the mixed signals, a noisy signal model can be considered a promotion of a noise-free mode[24]. Therefore, a noise-free linear instantaneous mixture ICA model is discussed. Similar to Eq (1), *N* unknown mutually statistically independent source signals are received by *M* sensors through an unknown linear channel transmission. The received signal formulated in Eq (1) can be simply expressed as

X(t)=AS(t) (4)

A matrix *W* should be obtained when ICA is used to process the received mixed signal to estimate the source signal:

Y(t)=WX(t)=WAS(t)=GS(t) (5)

where the ICA output *Y*(*t*) = [*y*_1_(*t*), *y*_2_(*t*), … *y*_*n*_(*t*)]^T^ is the estimation of unknown source signals *S*(*t*) = [*s*_1_(*t*), *s*_2_(*t*), … *s*_*N*_(*t*)]^T^, *W* is the de-mixing matrix (or weighted matrix), and *G* is the global matrix. If there is only one element approximately equal to 1 in each row and each column of *G* while the other elements are approximately zero, the source signals are successfully separated.

There are many approaches to solve the ICA problem using Eq (7) [25][26][27]. Since complex calculations must be solved in this study, a complex fast independent component analysis (cFastICA) algorithm [28][29] is applied, as proposed by Bingham and Hyvärinen from the Helsinki University of Technology in Finland based on the classic real fast ICA algorithm (FastICA)[30].

FastICA is one of the most common algorithms with a fast convergence, and the learning rate does not have to be set.

In the cFastICA algorithm, a more robust and faster approach is developed to approximate the negative entropy

$$J(y)\approx {\left[E\left\{F(y)\right\}-E\left\{F(v)\right\}\right]}^{2}$$ (6)

where *y* is the output variable with zero mean and unit variance; *v* is a Gaussian random variable with zero mean and unit variance; and *F*(⋅)is an arbitrary non-quadratic function.

The purpose of the algorithm is to maximize *J*(*y*) by selecting the mixing matrix *W*.

Similar to the FastICA algorithm, an approximate higher-order statistic (e.g., *F*(*y*) = −exp(−*y*^2^/2)) is applied to the above cost function in cFastICA. A derived iterative formula of the de-mixing matrix *W* is as follows:

$$\begin{array}{c} w_{k+1}=E\left\{X{\left(w_{k}^{H}X\right)}^{*}f(|w_{k}^{H}X{|}^{2})\right\}-E\left\{f(|w_{k}^{H}X{|}^{2})+|w_{k}^{H}X{|}^{2}f^{\prime }(|w_{k}^{H}X{|}^{2})\right\}w_{k}\\ w_{k+1}=\frac{w_{k+1}}{\left\|w_{k+1}\right\|} \end{array}$$ (7)

where *w* is a raw value of the de-mixing matrix *W* and *f*(⋅)and *f*′(⋅) are the first- and second-order derivatives of *F*(⋅), respectively.

By comparing Eqs (1) and (4), it is observed that the de-mixing matrix *W* of the cFastICA algorithm can be considered an array manifold of signals received by the antenna array.

As previously mentioned, if the global matrix *G* = *ŴÂ* has only one element that is approximately 1 in each row and each column and the other elements are approximately 0, the mixed signal will be successfully separated. Therefore, the inverse matrix (or pseudoinverse matrix when the number of sources *N* is not equal to the number of sensors *M*) *Â* of the de-mixing matrix estimation *Ŵ* can be considered the estimation of array manifold *A*. There are some differences in amplitude, phase and sort order between *Â* and *A* due to the inherent uncertainty of the ICA algorithm [22]. Fortunately, these differences do not affect the final DOA estimation results because the DOA depends on the ratio between the elements in a steering vector.

Certainly, the ICA algorithm should pre-process the received data, which includes centering and whitening the received data. These operations can improve the convergence properties, relieve the ill-posed problem, eliminate the information redundancy or reduce the effect of noise.

### 3.2 Estimating the DOA using the compressive sensing theory

CS theory is a new theory about sparse signal acquisition and recovery, which was proposed by Candes [31], Romberg [32], Tao and others in 2006. Unlike the traditional Nyquist sampling theorem, the CS theory is a new signal sampling, encoding and decoding theory that fully uses the signal sparsity or compressibility. The CS theory combines the sampling and compression into one. The realization process of the algorithm collects the non-adaptive linear projection measured value of the signal and reconstruct the original signal from a small number of measured values according to the corresponding reconstruction algorithm, which significantly reduces the sampling rate of signal. It can be used to accurately restore the original signal or estimate the signal parameters by using much less required measurement data than the classic Nyquist sampling theory.

In the CS theory, using a random measurement matrix, a sparse high-dimensional signal can be projected onto a low-dimensional space, and this random projection contains sufficient information to reconstruct the signal. Using the sparsity priori conditions of the signal, the original signal can be reconstructed by the optimization theory algorithm with a high probability.

As introduced in subsection 3.1, the weighted matrix estimation *Ŵ* is determined; then, the estimation *Â* = [*â*_1_,*â*_2,_…,*â*_*N*_] of the array manifold *A* is obtained. *â*_*n*_(*n* = 1, 2, …*N*) is the estimated steering vector with multipath component information and corresponds to each source signal. Thereafter, Eq (2) can be rewritten as

$$\begin{array}{lll} {\hat{a}}_{n} & = & \left[{\tilde{a}}_{n},\;{\tilde{a}}_{nL_{2}},{\tilde{a}}_{nL_{3}},\;\cdots ,{\tilde{a}}_{nL_{P}}\right]{\left[{\tilde{c}}_{nL_{1}},\;{\tilde{c}}_{nL_{2}},\;{\tilde{c}}_{nL_{3}},\;\cdots {\tilde{c}}_{nL_{p}}\right]}^{T}+\dot{n}\\ & = & BC+\dot{n} \end{array}$$ (8)

where $B=[{\tilde{a}}_{n},{\tilde{a}}_{nL_{1}},{\tilde{a}}_{nL_{2}},\cdots ,{\tilde{a}}_{nL_{P}}]$ is a set of steering vectors, which describe the direct component and multipath components, which are received from the same source and enter the array antenna. $C={\left[{\tilde{c}}_{nL_{1}},{\tilde{c}}_{nL_{2}},{\tilde{c}}_{nL_{3}},\cdots {\tilde{c}}_{nL_{P}}\right]}^{\text{T}}$ is a set of propagation attenuation coefficients of the multipath components relative to the direct components. In particular, ${\tilde{c}}_{nL_{1}}$ is the attenuation coefficient of the direct component, and its value is equal to 1. $\dot{n}$ is the discrepancy between the estimated steering vector (after adjusting the amplitude and sequence) and the actual steering vector caused by additive noise in Eq (1).

All components of a source signal can be extended to the entire space domain because a measured target in the entire space domain only occupies a small amount of angular resolution units, which implies that only the *P* non-zero elements in the entire space domain and remaining elements are zero. Therefore, the target distribution space is sparse. Consequently, the multi-objective estimation problem is considered a sparse vector reconfiguration, the non-zero elements of the sparse vector and their locations in the vector imply the magnitude and angle of the object.

According to the compressive sensing theory, the steering vector can be re-configured to an over-complete dictionary. The specific method samples the angular space or uniformly discretizes the angle space. As a result, the angle space is divided into uniform grids. Suppose the indices of *Q* grids are [*φ*_1_,*φ*_2_, …*φ*_*Q*_], these *Q* grids will be the candidate direction of the arrival vectors, and *P*⪡*Q*. According to Eq (5), the over-complete dictionary is *B* = [*b*(*φ*_1_),*b*(*φ*_2_), …*b*(*φ*_*Q*_) and $b(\varphi_{q})={\left[1,e^{-j2\pi d\text{sin}\varphi_{q}/\lambda },\cdots ,e^{-j2\pi (M-1)d\text{sin}\varphi_{q}/\lambda }\right]}^{\text{T}}$ (1≤*q*≤*Q*).

Eq (10) is a typical single-measurement vector (SMV) model in the compressive theory. Furthermore, it is an underdetermined equation, and the solution obtained using traditional methods is not unique. However, its most sparse solution can be found by solving the optimization problem

$$\text{min}{\left\|C\right\|}_{l_{0}}s.t.\;{\hat{a}}_{n}=BC+\dot{n}$$ (9)

where ${\left\|C\right\|}_{l_{0}}$ is the *l*_0_ norm of *C* or the number of non-zero elements of *C*. This is a non-deterministic polynomial-time hard problem (NP-Hard) [33]. There are $C_{M}^{P}$ possible linear combinations that satisfy Eq (11). A simpler *l*_1_ norm optimization can be used to solve this problem [34]. Thus, Eq (1) can be expressed as

$$\text{min}{\left\|C\right\|}_{l_{1}}s.t.\;{\hat{a}}_{n}=BC+\dot{n}$$ (10)

This noisy SMV problem can be converted to solve the second-order cone programming (SOCP) [35]:

$$\text{min}{\left\|C\right\|}_{l_{1}}s.t.\;{\left\|{\hat{a}}_{n}-BC\right\|}_{l_{2}}^{2}\leq \beta^{2}$$ (11)

where *β* is the variance of $\dot{n}$. In practice, after acquiring and tracking the GNSS signal, the discrepancy between the local generated signal and the practical received signal separated by ICA can be obtained. Meanwhile, the discrepancy between the estimated steering vector (after adjusting the amplitude and sequence) and the actual steering vector can be obtained.

In this study, the interior point method (IPM) is applied [36] to solve Eq (11) and achieve the sparse spectrum of *C*.

With reference to Eq (8), the following conclusion can be made. For a signal source with a direct component and a multipath component, if *φ*_*q*_ is equal to the DOA of the direct component, the attenuation coefficient *c*_*q*_ is approximately equal to 1. If *φ*_*q*_ is equal to the multipath component DOA, the attenuation coefficient *c*_*q*_ is approximately equal to the attenuation coefficient of this component. If *φ*_*q*_ is not equal to the direct component or multipath component DOA, the corresponding *c*_*q*_ is approximately equal to 0. Therefore, expected results can be obtained through the sparse spectrum peak. Additionally, the incident signal can be determined from the direct or multipath component based on the value of *c*_*q*_.

### 3.3. DOA estimation algorithm summarization

**Fig 1:**
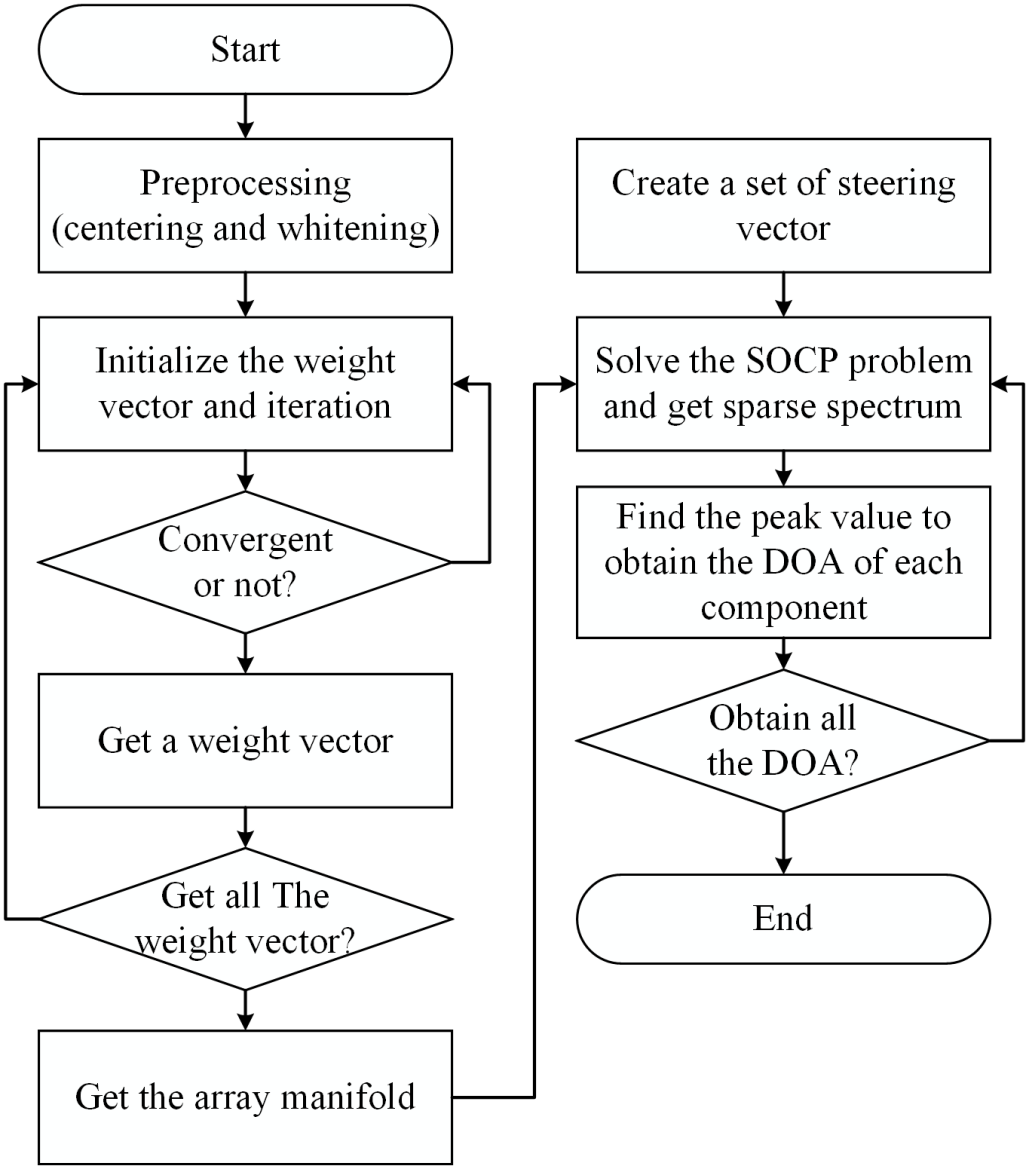
Flow chart of the proposed algorithm.

The proposed DOA estimation algorithm is summarized as follows, and a flow chart of the algorithm is shown in Fig 1.

1. Pre-process received array signals, including the centering and whitening processes.
2. Initialize the weighted vector *w* of the first component and successively iterate *w* using Eq (7).
3. If the algorithm does not converge, go to step (2); if the algorithm converges, obtain the weight vector of an independent component.
4. Obtain the weight vectors of all components, which is the de-mixing matrix *Ŵ*.
5. Calculate the inverse (or pseudoinverse) of the de-mixing matrix *Ŵ* to obtain the array manifold estimation *Â*.
6. Create an over-complete dictionary *B* according to the grid indices of the DOA space.
7. Solve the SOCP problem and obtain the sparse spectrum of the propagation attenuation coefficient set *Ĉ* for a source signal.
8. Find the peak values of the sparse spectrum and obtain the DOAs of the direct and multipath components that correspond to *â*_*n*_.
9. Obtain all DOAs of the direct and multipath components one-by-one.

## 4. Test results and analysis

This section focuses on evaluating the proposed coherent signal DOA estimation scheme. As introduced in Section 1, currently, the DOA estimation methods of coherent signals are mainly spatial smoothing techniques such as SS-MUSIC and SS-ESPRIT, which are widely accepted. In addition, [37] provides a detailed comparison of these two methods. Correspondingly, SS-MUSIC is significantly advantageous. Therefore, the proposed ICA-CS and SS-MUSIC schemes are compared in this section.

To comprehensively assess ICA-CS, four sets of simulation studies were performed to verify the spatial resolution, accuracy at different SNRs, snapshots and underdetermined conditions.

Simulation conditions were set up based on assumptions of the actual GNSS signal transmission environment. The processing object is a GNSS signal in this paper, which indicates that at least four satellites are required to complete the positioning solution, so the direct signals are set from four different directions. To study the adaptability of the simulation, for the source signals, we set the existence of one multipath component, two multipath components and no multipath component. The following general simulation conditions are shown in Table 1. The GNSS navigation signals from 4 satellites are incident to the uniform linear array of 12 elements from different angles, the incident angles are -40°, -20°, 10°, and 40°. There are 2 multipath components for the first signal, and the incident angles are 30° and -10°; the second and third signals each have 1 multipath component, and the incident angles are -30° and 20°; and the fourth signal does not have a multipath component. The propagation attenuation coefficients of the 4 multipath components are -6 dB, -7 dB, -8 dB, and -9 dB.

**Table 1:** The general simulation conditions.

Signal type	GNSS navigation signals			
**Number of satellites**	4			
**Incident angles of direct components**	-40°	-20°	10°	40°
**Number of multipath components**	2	1	1	0
**Incident angles of multipath components**	30°, -10°	-30°	20°	NA
**Propagation attenuation coefficients**	-6 dB, -9 dB	-7 dB	-8 dB	NA

### Simulation 1: Spatial resolution

**Fig 2:**
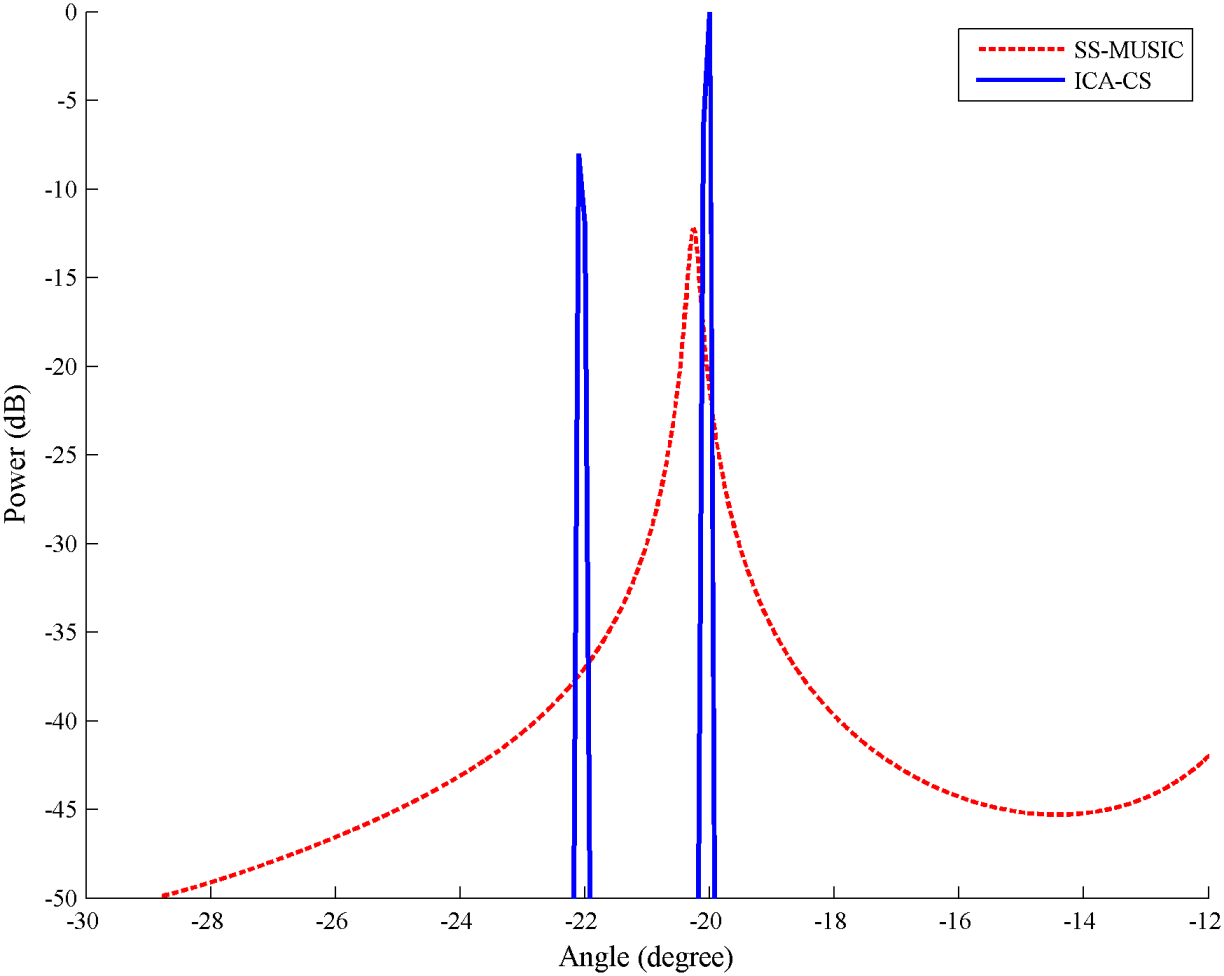
Local contrast diagram of spatial spectrum of the independent component analysis-compressive sensing and spatial smoothing-multiple signal classification algorithm.

For Simulation 1, the signal-to-noise ratio (SNR) of the signals was 20 dB, and the sampling number was 2000. The incident angle of multipath component for the second signal was set as -22°. The space spectrum estimation results of the two algorithms are shown in Fig 2.

From the simulation results, the ICA-CS algorithm has sharper peaks than SS-MUSIC; when the direct component and its multipath component are separated by 2 degrees, the SS-MUSIC algorithm cannot distinguish their arrival directions, and the ICS-CS algorithm can obtain the desired result. Thus, the DOA estimation results of the ICA-CS algorithm have a higher resolution in the spatial spectrum because the spatial spectrum of the MUSIC algorithm reflects the distance between the steering vector and noise subspace, and the performance of the algorithm depends on their orthogonality. In the coherent signal reception environment, a distorted covariance matrix worsens the orthogonality. Correspondingly, the resolution of the MUSIC algorithm deteriorates. The spatial spectrum of the ICA-CS reflects the signal energy from a certain angle in the space domain, and the spatial spectrum amplitude is zero at the angle of no signal arrival; therefore, needle-shaped curves appear in Fig 2.

**Fig 3:**
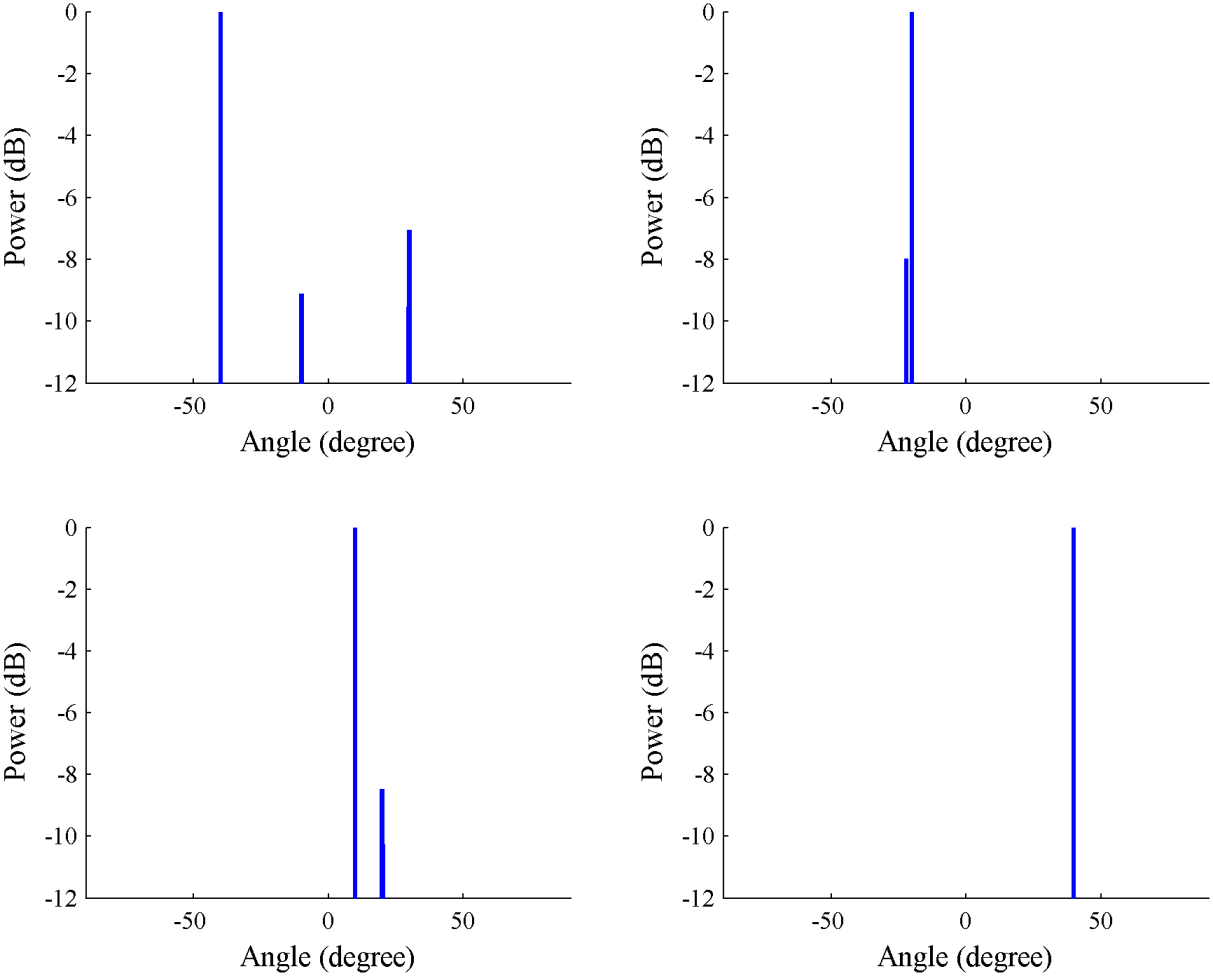
Spatial spectrum of all components for each signal using the independent component analysis-compressive sensing algorithm.

In addition, the DOAs of all components can be achieved from the spatial spectrum of the array signal using the SS-MUSIC algorithm. However, it is difficult to distinguish the direct and multipath components received from the same source signal. The ICA-CS algorithm solves the steering vector that corresponds to each source signal to calculate the DOAs of its direct and multipath components. As a result, the spatial spectrums of the four source signals are shown in Fig 3. Different multipath components and their attenuation are also observed.

### Simulation 2: DOA estimation error at various SNRs

**Fig 4:**
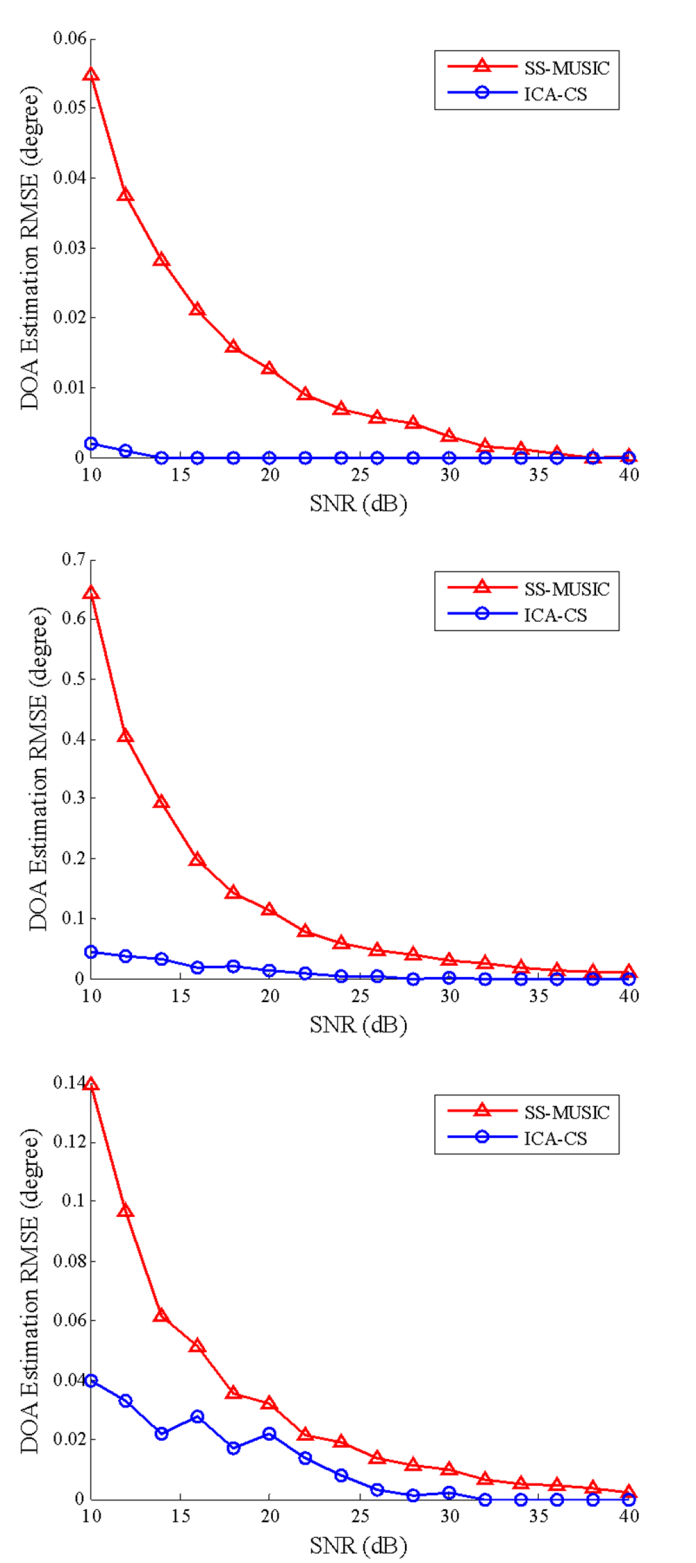
DOA estimation root-mean-square errors of all components at various signal-to-noise ratios.

The conditions of Simulation 1, except for the SNR, were used to assess the DOA estimation error at various SNRs. The range of tested SNR is 10–40 dB with an interval of 2 dB. The estimated DOA errors were obtained using 100 independent trials of the experiment. A different computer realization of the noise was rendered for each trial. For the first source signal, the root-mean-square error (RMSE) of the DOA estimation of the direct and multipath components changes with the SNR for different algorithms, as shown in Fig 4 (the three graphs show the estimation results of the direct component, multipath component 1, and multipath component 2).

Fig 4 shows that the error of the two algorithms gradually decreases to zero with increasing SNR. A larger SNR results in a smaller DOA RMSE. Whether it is a direct component or two multipath components, the introduced ICA-CS outperforms the SS-MUSIC algorithm. In particular, an expected DOA estimation is obtained using the ICA-CS at low SNR.

### Simulation 3: DOA estimation error in various snapshots

**Fig 5:**
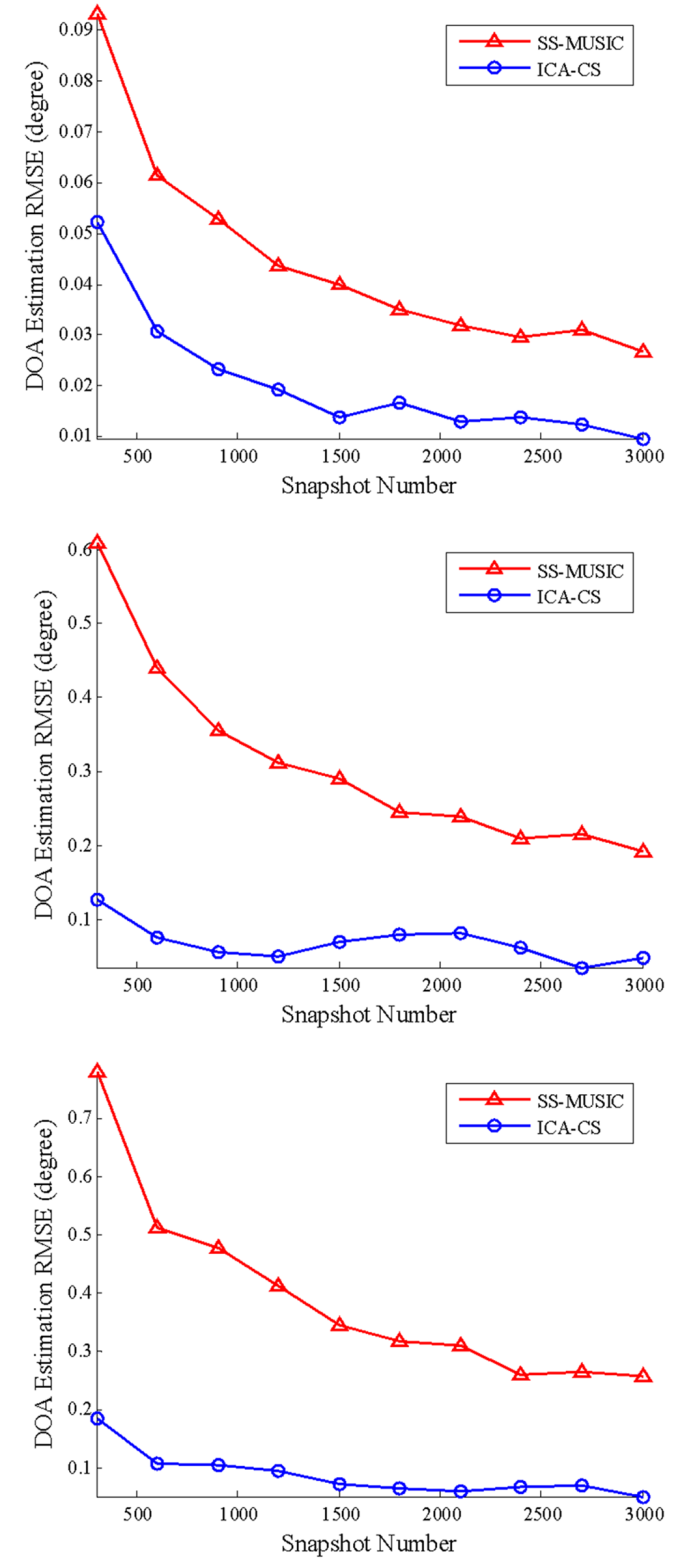
DOA estimation root-mean-square errors of all components in various snapshots.

In the following, the conditions of Simulation 1, except the snapshot, were used to assess the DOA estimation error in various snapshots. In this test, the snapshot changes from 300 to 3000. The SNR is equal to 10 dB. In total, 100 independent trials of the experiment were conducted. The RMSEs of the DOA estimation versus the number of snapshots are plotted in Fig 5 (the three graphs show the direct component, multipath component 1, and multipath component 2).

Fig 5 shows a similar DOA estimation RMSE behavior to simulation 2. The DOA estimation RMSEs gradually decrease when the number of snapshots increases. The DOA estimation errors of the direct and multipath components are less than 0.2° for the lowest number of snapshots using the ICA-CS algorithm. For the SS-MUSIC algorithm, whether for a direct component or two multipath components, its performance is significantly worse than the ICA-CS algorithm.

In Simulations 2 and 3, since the DOA estimation performance of the SS-MUSIC algorithm depends on the covariance matrix, and the low SNR and low snapshot conditions lead to covariance, a larger error occurs and creates a large distance error between the steering vector and the noise subspace. Additionally, the spatial smoothing algorithm reduces the array aperture and further decreases the estimation accuracy for the same number of antenna array elements. The ICA-CS algorithm does not destroy the signal details and de-noise the input signals, which can help recover the actual steering vector with a small amount of error.

### Simulation 4: ICA-CS in underdetermined case

In the SS-MUSIC algorithm, the spatial smoothing will deteriorate the array aperture, which implies that the signal from at least 1 element is lost after the spatial smoothing process [3]. In addition, the MUSIC algorithm requires that the number of array elements *M* is greater than the incident signal number *N*×*P* [1]. Therefore, the DOA estimation of *N*×*P* incident signals (including all direct and multipath components) requires at least (*N*×*P+2*) array elements if the SS-MUSIC algorithm is applied.

The new DOA estimation method ICA-CS can be used on the condition that the number of array elements *M* is not less than the number of source signals *N*. In CS theory, the number to carry out sparse signal recovery is as large as half the sensor number [38]; that is, the number of multipath components for each source signal *P* is not greater than *M*/2. Therefore, the ICA-CS algorithm can be used in the underdetermined case where *N*≤*M* and *P*≤*M*/2, that is, the proposed algorithm can be used to perform the DOA estimation of *M* source signals with *N*×*P* = *M*^2^/2 components.

**Fig 6:**
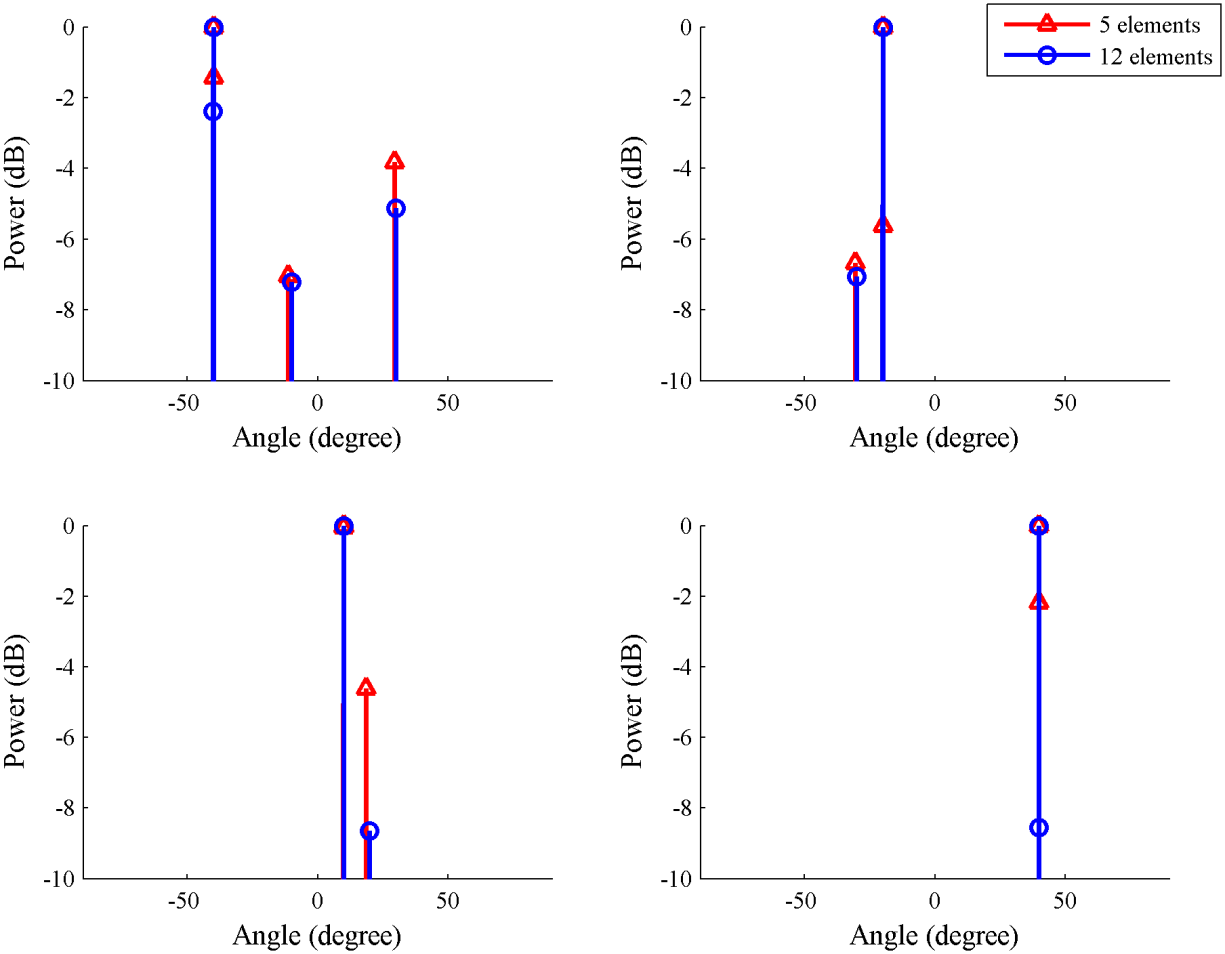
Contrast diagram of spatial spectrum for each signal for 5 and 12 array elements using the independent component analysis-compressive sensing algorithm.

In this simulation, the number of antenna array elements was set to 5. Under this condition, the SS-MUSIC algorithm does not perform well. Therefore, we compare the estimation results using the ICA-CS algorithm when the element number is 5 and 12. The other conditions are consistent with Simulation 1. Fig 6 shows the contrast diagram of the spatial spectrum for 4 source signals under two different element number settings.

When the proposed algorithm realizes the DOA estimation of a multipath signal in the underdetermined condition, or if the number of antenna array elements is less than the number of received signals, the simulation performance decreases, and the maximum estimation error is approximately 1°.

## 5. Conclusion

In this paper, we proposed a multipath DOA estimation method, which combines the complex fast independent component analysis with the compressed sensing theory without decoherence processing. The simulation results illustrate the effectiveness of the proposed method. When the SNR is 10 dB, the RMSE is maintained below 0.05°, which is obviously better than the classic SS-MUSIC algorithm. When the SNR is 10 dB and the sampling number is only 300, the estimated DOA RMSE can be maintained below 0.2°, which is also applicable in the absence of sufficient data. This algorithm can also perform a DOA estimation for multipath signals in underdetermined cases that cannot be processed by other algorithms.

Similar to [14], a basis mismatch [39] may occur when the bearings of the far-field sources appear between discretized angles. Luckily, the bearing space was discretized into a grid of 0.1° in the simulations of this paper, which makes the performance degradation errors because of the basis mismatch less than 0.1°, which is the resolution in simulations. In other words, the simulation results are obtained in the presence of the basis mismatch. If the basis mismatch problem is solved, the DOA estimation performance will be better, and this issue will be one of my future studies.

Because of the experimental conditions are relatively complex, for example, to satisfy the test requirements, we require several GNSS antennas and set them as a uniform linear array while ensuring that specified number of direct components and its corresponding multipath components are received by the antenna array. Thus, we only make the software simulations and will perform the practical experiment in future studies.

## Supporting information

S1 Fig Flow chart of the proposed algorithm. (TIF)

S2 Fig Local contrast diagram of spatial spectrum of the independent component analysis-compressive sensing and spatial smoothing-multiple signal classification algorithm. (TIF)

S3 Fig Spatial spectrum of all components for each signal using the independent component analysis-compressive sensing algorithm. (TIF)

S4 Fig DOA estimation root-mean-square errors of all components at various signal-to-noise ratios. (TIF)

S5 Fig DOA estimation root-mean-square errors of all components in various snapshots. (TIF)

S6 Fig Contrast diagram of spatial spectrum for each signal for 5 and 12 array elements using the independent component analysis-compressive sensing algorithm. (TIF)
