# Supplementary figures and images for: Direction-of-arrival estimation of multipath signals using independent component analysis and compressive sensing
Source: PLoS One. 2017 Jul 27;12(7):e0181838. doi: 10.1371/journal.pone.0181838 (PMC5531488; doi:10.1371/journal.pone.0181838)

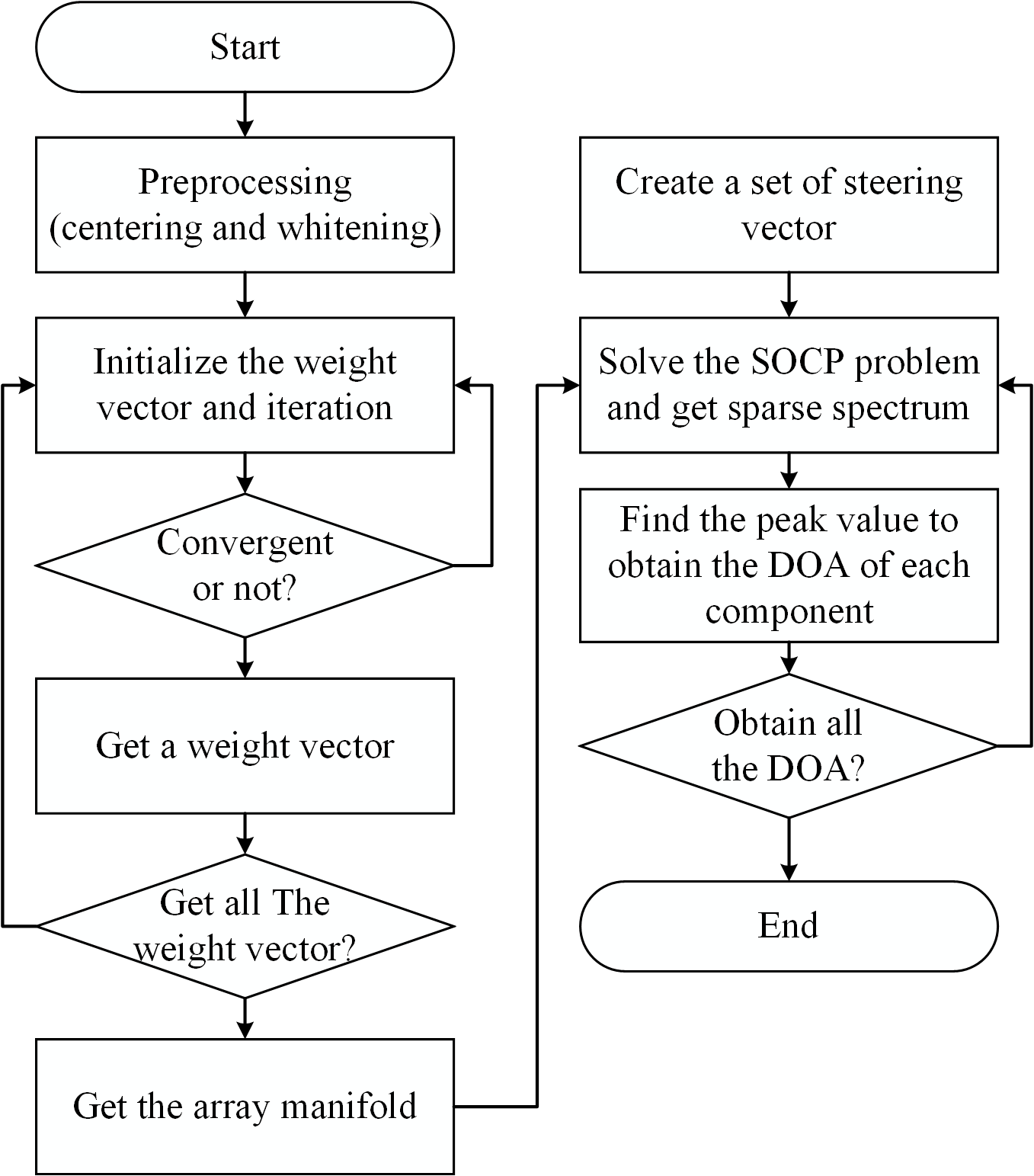

Supplement: S1 Fig — (TIF) [file pone.0181838.s001.tif]

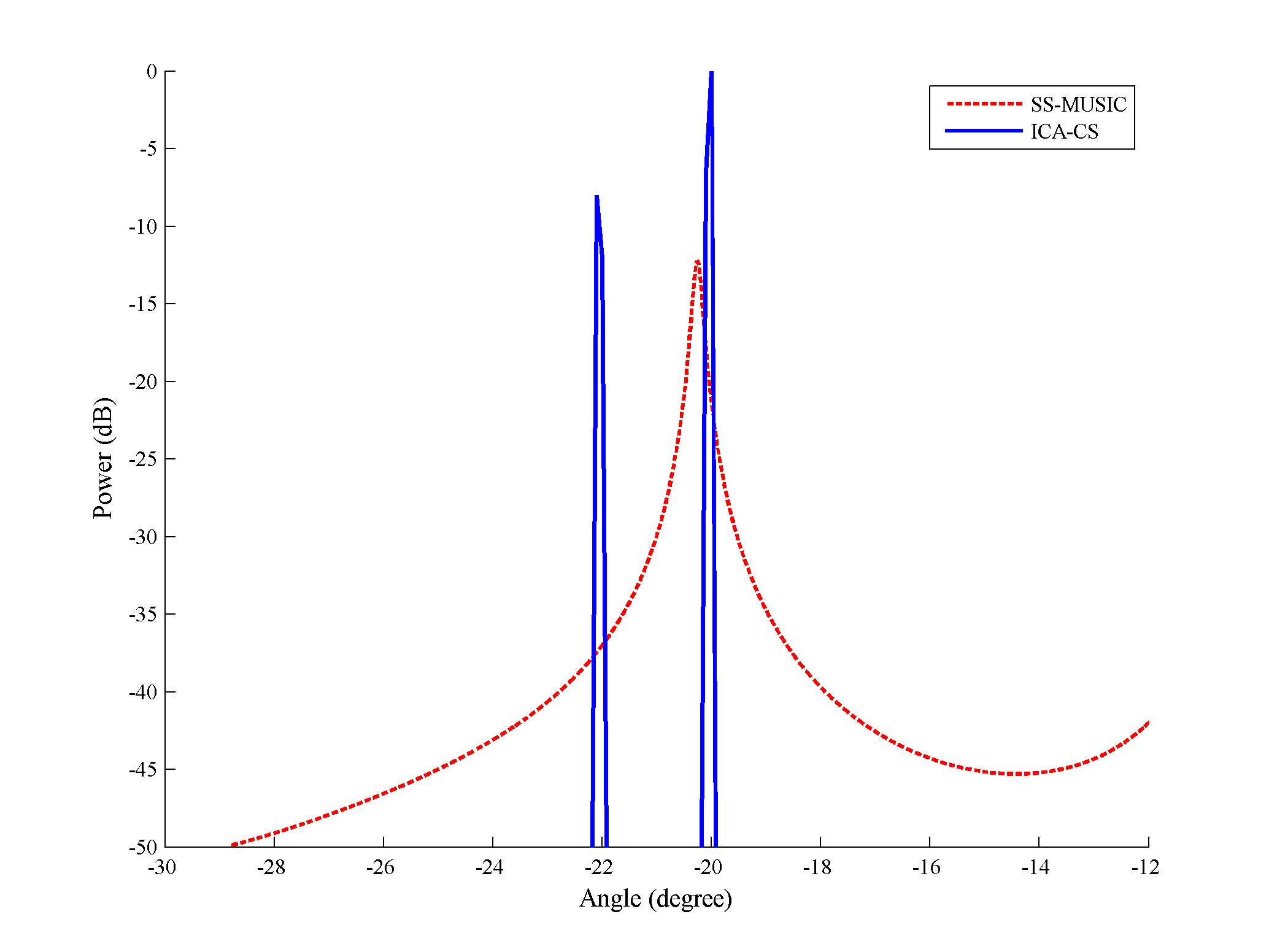

Supplement: S2 Fig — (TIF) [file pone.0181838.s002.tif]

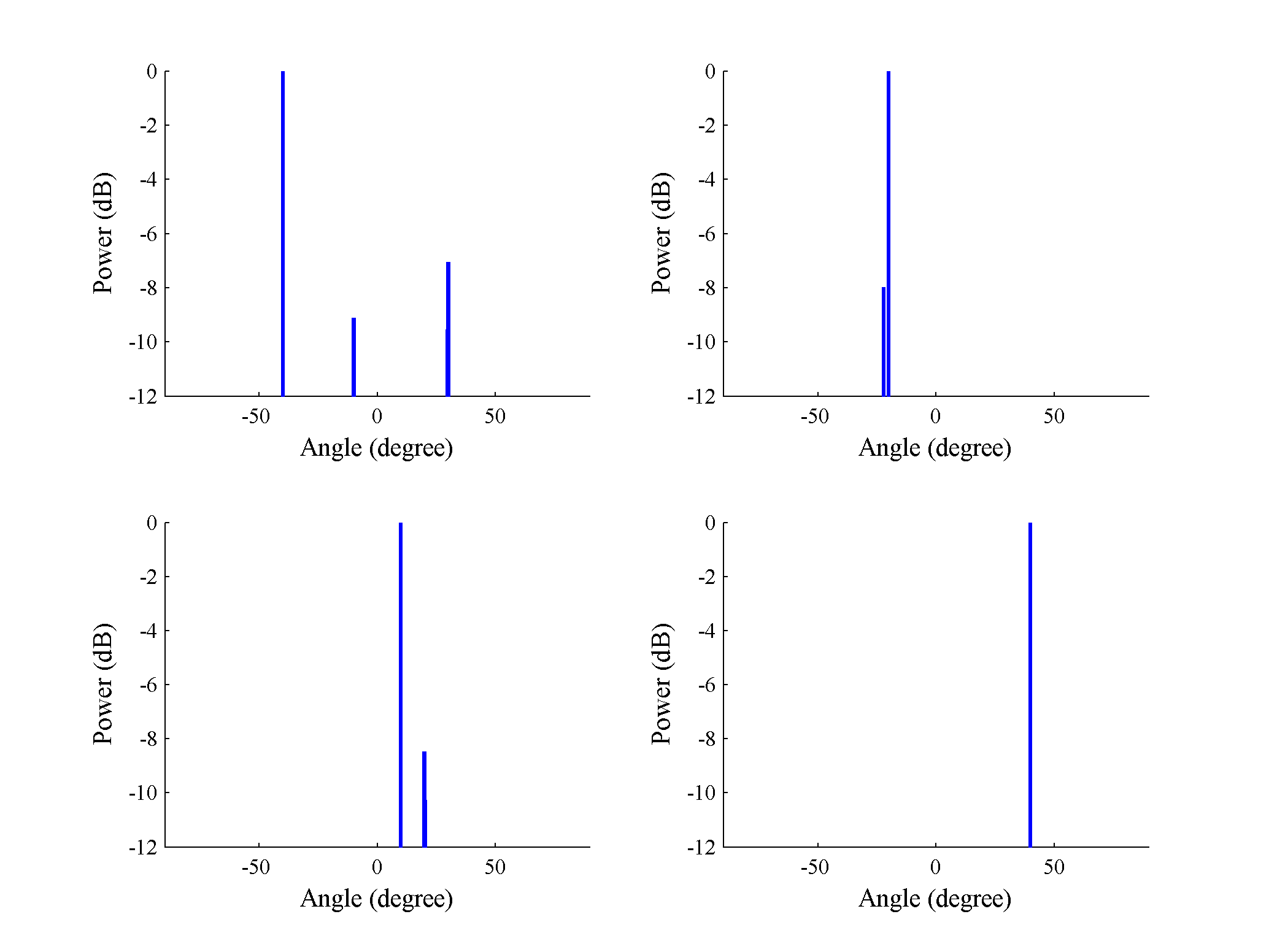

Supplement: S3 Fig — (TIF) [file pone.0181838.s003.tif]

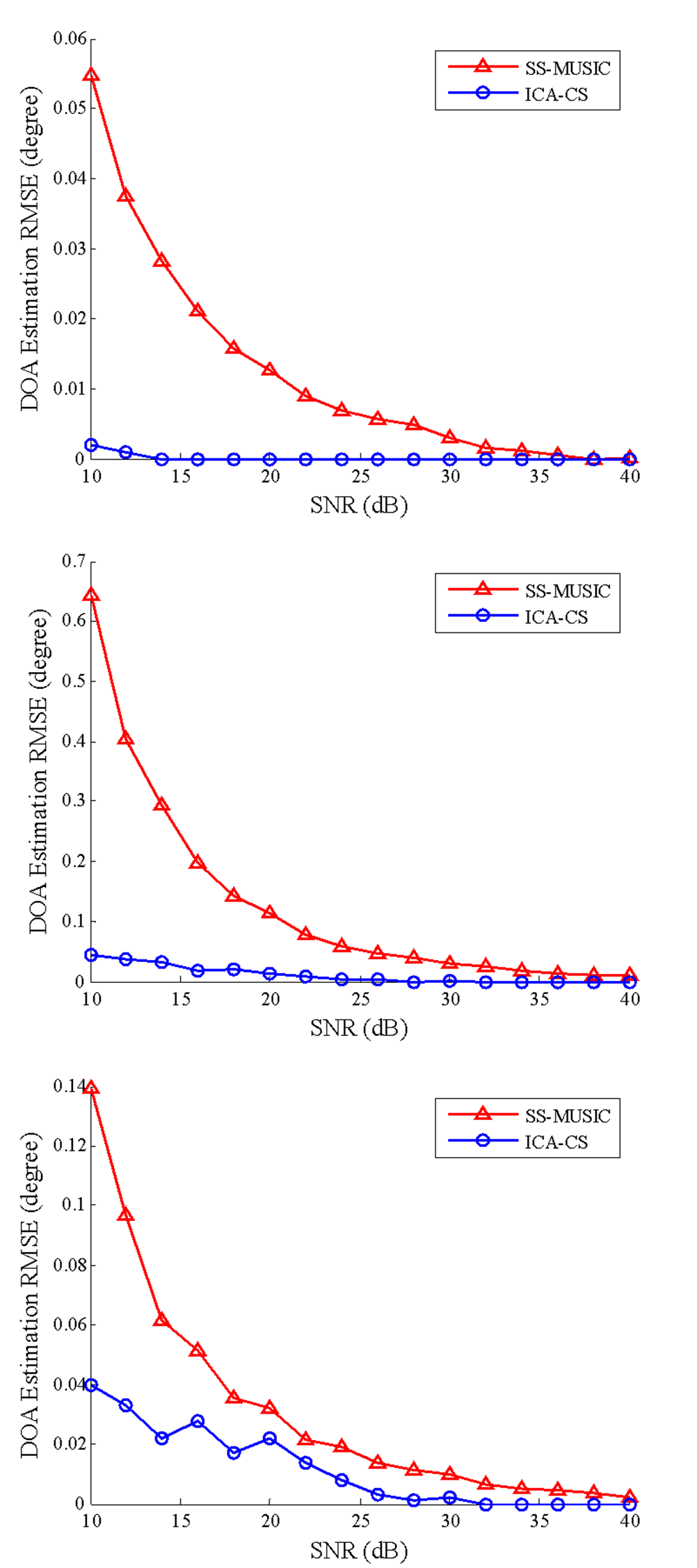

Supplement: S4 Fig — (TIF) [file pone.0181838.s004.tif]

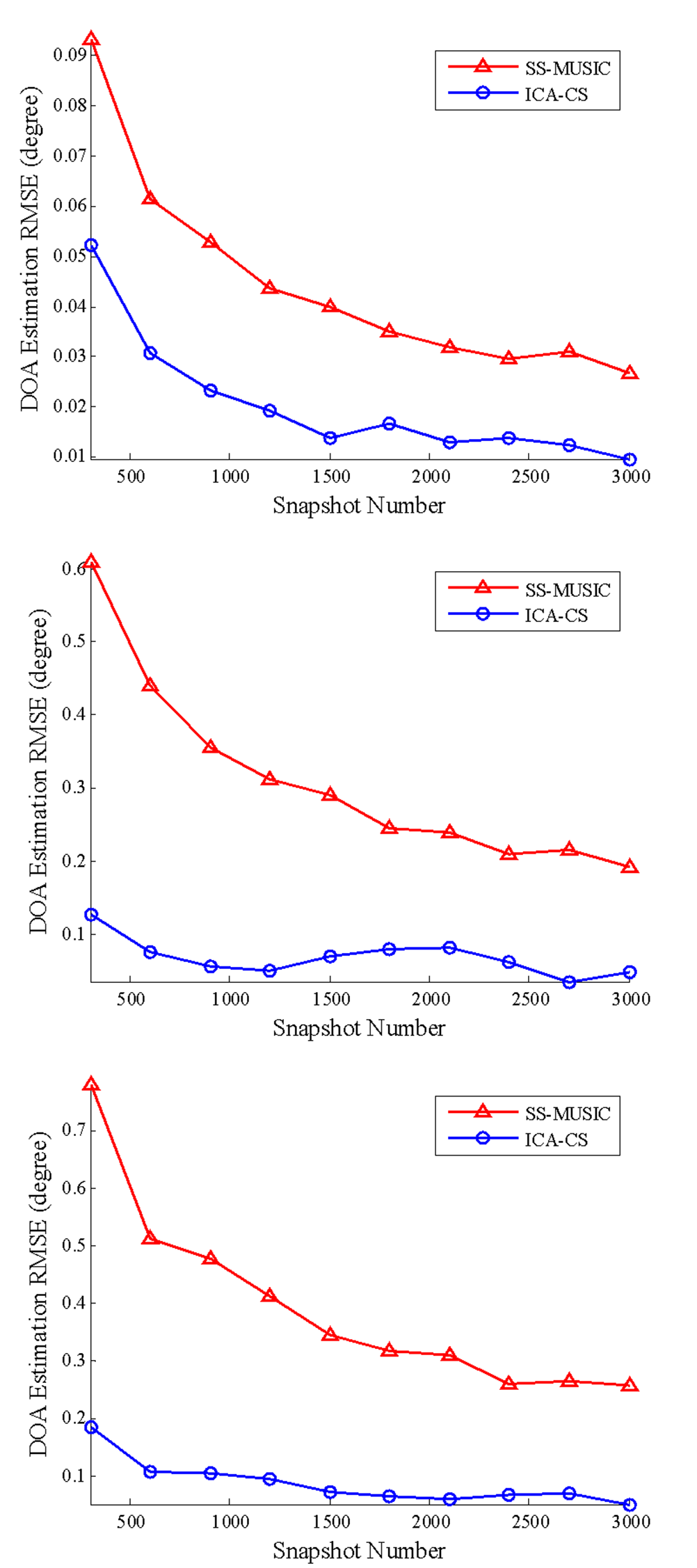

Supplement: S5 Fig — (TIF) [file pone.0181838.s005.tif]

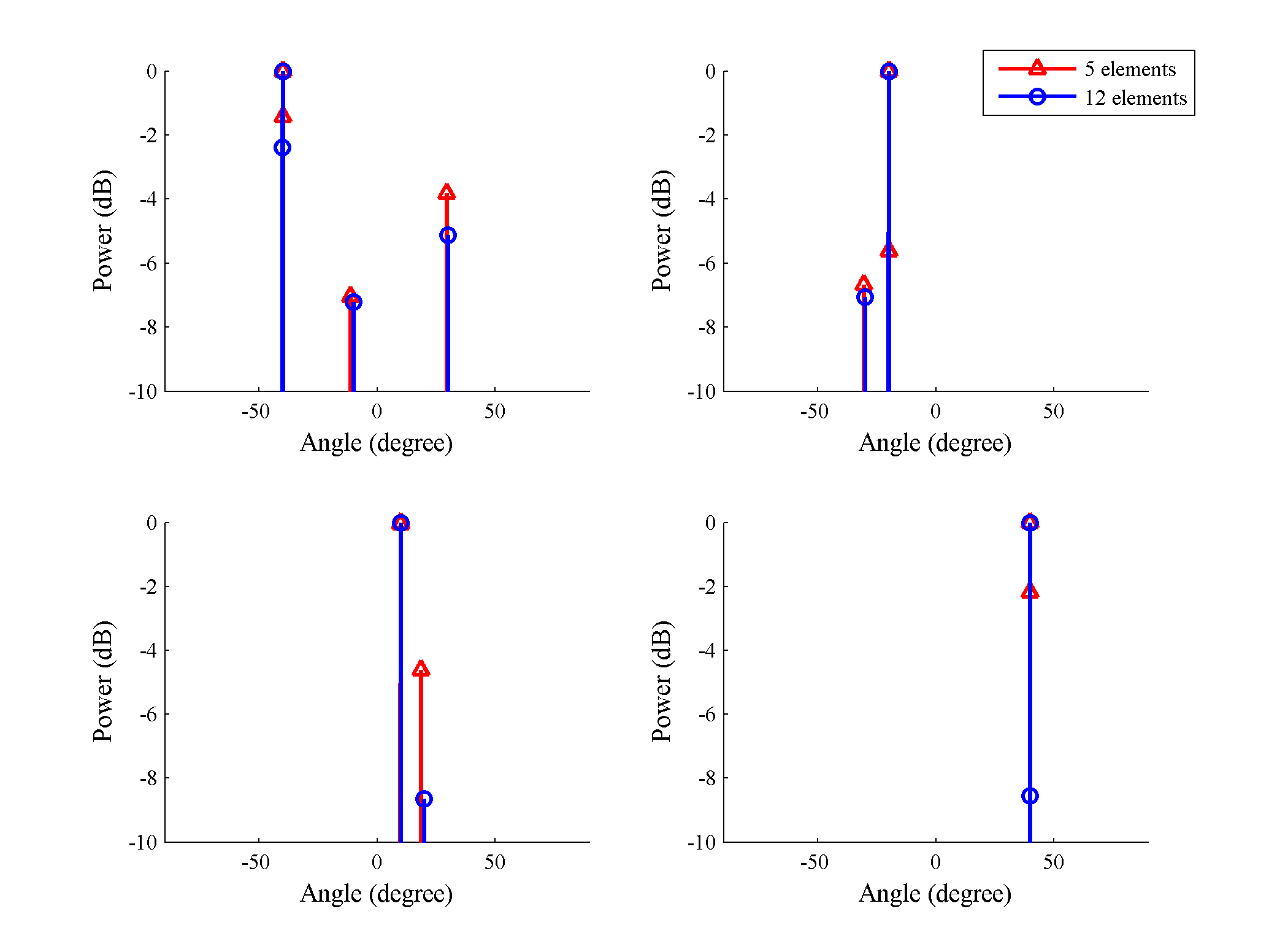

Supplement: S6 Fig — (TIF) [file pone.0181838.s006.tif]
